# A panel of recombinant proteins from human-infective *Plasmodium* species for serological surveillance

**DOI:** 10.1186/s12936-020-3111-5

**Published:** 2020-01-17

**Authors:** Nicole Müller-Sienerth, Jarrod Shilts, Khamisah Abdul Kadir, Victor Yman, Manijeh Vafa Homann, Muhammad Asghar, Billy Ngasala, Balbir Singh, Anna Färnert, Gavin J. Wright

**Affiliations:** 1 Cell Surface Signalling Laboratory, Wellcome Sanger Institute, Cambridge, UK; 2 Malaria Research Centre, Universiti Malaysia Sarawak, Kota Samarahan, Sarawak Malaysia; 3 Division of Infectious Diseases, Department of Medicine Solna, Karolinska Institutet, Stockholm, Sweden; 4 Department of Parasitology and Medical Entomology, Muhimbili University of Health and Allied Sciences, Dar es Salaam, Tanzania; 5 Department of Women’s and Children’s Health, International Maternal and Child Health (IMCH), Uppsala University, Uppsala, Sweden; 6 Department of Infectious Diseases, Karolinska University Hospital, Stockholm, Sweden

**Keywords:** *Plasmodium*, Serology, Antigen, Recombinant protein, Antibody, Malaria

## Abstract

**Background:**

Malaria remains a global health problem and accurate surveillance of *Plasmodium* parasites that are responsible for this disease is required to guide the most effective distribution of control measures. Serological surveillance will be particularly important in areas of low or periodic transmission because patient antibody responses can provide a measure of historical exposure. While methods for detecting host antibody responses to *Plasmodium falciparum* and *Plasmodium vivax* are well established, development of serological assays for *Plasmodium knowlesi*, *Plasmodium ovale* and *Plasmodium malariae* have been inhibited by a lack of immunodiagnostic candidates due to the limited availability of genomic information.

**Methods:**

Using the recently completed genome sequences from *P. malariae*, *P. ovale* and *P. knowlesi,* a set of 33 candidate cell surface and secreted blood-stage antigens was selected and expressed in a recombinant form using a mammalian expression system. These proteins were added to an existing panel of antigens from *P. falciparum* and *P. vivax* and the immunoreactivity of IgG, IgM and IgA immunoglobulins from individuals diagnosed with infections to each of the five different *Plasmodium* species was evaluated by ELISA. Logistic regression modelling was used to quantify the ability of the responses to determine prior exposure to the different *Plasmodium* species.

**Results:**

Using sera from European travellers with diagnosed *Plasmodium* infections, antigens showing species-specific immunoreactivity were identified to select a panel of 22 proteins from five *Plasmodium* species for serological profiling. The immunoreactivity to the antigens in the panel of sera taken from travellers and individuals living in malaria-endemic regions with diagnosed infections showed moderate power to predict infections by each species, including *P. ovale*, *P. malariae* and *P. knowlesi.* Using a larger set of patient samples and logistic regression modelling it was shown that exposure to *P. knowlesi* could be accurately detected (AUC = 91%) using an antigen panel consisting of the *P. knowlesi* orthologues of MSP10, P12 and P38.

**Conclusions:**

Using the recent availability of genome sequences to all human-infective *Plasmodium* spp. parasites and a method of expressing *Plasmodium* proteins in a secreted functional form, an antigen panel has been compiled that will be useful to determine exposure to these parasites.

## Background

Malaria is an infectious disease that remains a global health problem causing an estimated 219 million clinical cases resulting in 435,000 deaths in 2017 [1]. The disease is caused by parasites of the genus *Plasmodium* and several species are known to regularly infect humans. The vast majority of deaths occur in sub-Saharan Africa and are caused by *Plasmodium falciparum*, whereas outside of Africa, *Plasmodium vivax* is responsible for over half of all malaria infections leading to significant morbidity and mortality [2]. Much less is known about the other human-infective *Plasmodium* species, *Plasmodium malariae*, *Plasmodium ovale* and *Plasmodium knowlesi* both in terms of their global distribution and clinical impact. *Plasmodium knowlesi*, a parasite typically found in macaques, is a significant cause of human malaria in Southeast Asia, causing a spectrum of disease ranging from mild to fatal infections [3]. Malaysia has the highest incidence of *P. knowlesi* malaria with over 6700 cases reported in the last 2 years compared to only 85 cases of indigenous human malaria (unpublished data from the Ministry of Health, Malaysia).

Diagnosis of *Plasmodium* infections and epidemiological surveillance is important for guiding the distribution of resources into intervention measures and establishing their clinical impact over time [4]. Methods to measure the prevalence of *Plasmodium* infections include microscopy, rapid diagnostic tests (RDTs) and PCR-based approaches, each differing in their sensitivity, infrastructure requirements, and ability to diagnose the different species. Serological assays can provide a historical record of infection and because of the specificity of antibody-antigen binding, could also potentially discriminate between different *Plasmodium* spp. infections. Host antibodies appear rapidly after initial infection and can persist for months and even years after the parasites have been cleared [5, 6]. Serological screening has been applied in epidemiological settings to detect parasite exposure, evaluate transmission trends of malaria [7–10], and identify antibody-based correlates of protection [11, 12]. It is also used in blood donation centres, where, due to the increase in international travel and migration, the need for serological diagnosis is becoming more important to reduce the risk of transfusion-transmitted infections. Currently, many centres assess these risks using patient questionnaires which is generally unsatisfactory; moreover, the limitations and costs of the currently available serological tests often make implementing these assays economically unattractive [13].

Many antibodies recognise epitopes that are only formed in the context of an antigen in its native conformation [14]. To detect these antibodies, it is vitally important that the proteins used are correctly folded so that they faithfully form these epitopes. Expressing *Plasmodium* proteins in a soluble recombinant form has proved challenging, perhaps because of the high A:T content of the genome and lack of recognisable protein domains in many *Plasmodium* proteins [15]. This problem is especially acute for parasite proteins that are secreted or embedded in membranes because these proteins additionally contain structurally critical post-translational modifications, such as disulfide bonds that are not typically added by many commonly used expression systems. Recently, a method of expressing large panels of recombinant *Plasmodium* proteins was developed that retained many of their biochemical functions [16]. Central to this approach was the use of a mammalian expression system which increases the chances that appropriate post-translational modifications are correctly added to ensure proteins adopt their native fold. For antigens expressed using this method a large fraction—and in some cases all—the immunoreactivity to antigens was heat-labile, demonstrating that antibodies that recognise conformational epitopes represent a major component of the humoral response [16]. Previously, this approach was used to create libraries of soluble recombinant merozoite cell surface and secreted proteins that encompass the entire ectodomain from both *P. falciparum* [16, 17] and *P. vivax* [18]. Using sera from patients living in endemic regions, several of these proteins were found to be highly immunoreactive and might therefore be useful target antigens in serological assays [8, 12]. Expanding the antigen panel to include the other parasite species infecting humans would be especially valuable if they could be used to determine exposure to the different species of *Plasmodium*. Here, the recent availability of high-quality genome sequences from the three other human-infective *Plasmodium* parasites: *P. knowlesi*, *P. ovale* and *P. malariae* were used to extend the available panel of proteins and were tested for reactivity to sera from individuals infected with different *Plasmodium* parasites.

## Methods

### Study populations

Collection of sera from Malawian adults that were previously used to determine its effectiveness as an adjunct therapy to treat cerebral malaria was approved by the National Health Sciences Research Committee of Malawi [19]. Plasma from adult travellers, microscopy-diagnosed with malaria and species further confirmed by multiplex PCR for all species except *P. knowlesi* [20] after returning from visits to malaria-endemic regions were obtained from the Karolinska University Hospital, Stockholm, Sweden (*n* = 81). Of these, 53 were from travellers of European origin and 28 from travellers born in malaria-endemic regions as follows: Angola (3 individuals), Burundi (1), Cameroon (1), Democratic Republic of the Congo (1), Eritrea (4), Ethiopia (1), The Gambia (1), India (3), Ivory Coast (2), Kenya (5), Pakistan (1), Thailand (1), and Uganda (4). Plasma from an endemic region with diagnosed infections were from a longitudinally followed population from Nyamisati, in the Rufiji region, Tanzania (*n* = 21) in 1994 when transmission was high [21]. Swedish residents with no history of visiting malaria-endemic countries were included as controls (*n* = 28). Ethical approval was granted by the Ethical Review Board of the National Institute for Medical Research in Tanzania, and the Regional Ethical Review Board in Stockholm, Sweden (Dnr. 00-084, 2012/1151-32, 2006/893-31/4, 2018/2354-32). Serum samples from adult malaria patients with PCR-confirmed *P. knowlesi* mono-infection were collected at Kapit Hospital in Malaysian Borneo (*n* = 50) and from uninfected adult controls from Kapit Division (*n* = 66) after informed consent had been obtained. Approval to conduct this study was obtained from the Medical Research and Ethics Committee of the Ministry of Health, Malaysia and the Medical Ethics Committee, Faculty of Medicine and Health Sciences, Universiti Malaysia Sarawak.

### Recombinant protein construct design, expression and manipulation

The orthologues of immunoreactive blood-stage antigens from *P. knowlesi*, *P. malariae* and *P. ovale* were identified from their respective genome sequences [22–24]. For *P. ovale*, proteins from *Plasmodium ovale curtisi* were selected since the manual gene annotation of this genome resulted in complete open reading frames compared to the draft genome available for *Plasmodium ovale wallikeri* [24]. Sequences corresponding to the entire ectodomains were identified using software tools to predict the location of the signal peptides, GPI-anchor and transmembrane regions [25, 26]. In some cases, for example, *P. malariae* P38, this analysis helped improve automated gene prediction by identifying missing signal peptides. Based on these predictions, the ectodomain regions were determined by removing signal sequences and transmembrane domains. All potential N-linked glycosylation sites were systematically mutated by substituting the serine/threonine in the context of an N-linked glycosylation sequon for alanine to prevent inappropriate glycosylation when expressed in mammalian cells as described previously [16]. All sequences were codon-optimized for expression in human cells, flanked with unique 5′ NotI and 3′ AscI restriction enzyme sites to allow inframe cloning in a plasmid containing a highly efficient mouse variable κ light chain signal peptide [27] and a rat Cd4d3+ 4 epitope tag followed by either a peptide sequence allowing enzymatic biotinylation and/or 6-his tag for purification [28]. Proteins were expressed by transient transfection in suspension-grown HEK293E [29] and HEK293-6E cells [30], essentially as described previously [31]. In brief, HEK293 cells were seeded the day prior to transfection at a density of 2.5 × 10^5^ cells mL^−1^ (HEK293E) or 1.0 × 10^6^ cells mL^−1^ (HEK293-6E). Cells are routinely cultured in volumes of 50 mL in Freestyle293 media following the manufacturer’s recommendations; for HEK293E cells, the media is supplemented with 1% FCS. To ensure efficient biotinylation, the cell culture media used to produce bait proteins with D-biotin to a final concentration of 100 μM. The following day, cells were transfected as described [31] using either 25 μg (HEK293E) or 50 μg (HEK293-6E) of the bait plasmid constructs. To enzymatically monobiotinylate proteins, cells were co-transfected with a plasmid encoding a secreted version of the *Escherichia coli* BirA enzyme (Addgene plasmid number 64395), at a 10:1 ratio, essentially as described [31]. Cultures were harvested 6 (HEK293E) or 5 (HEK293-6E) days post-transfection by first pelleting the cells by centrifugation at 3000×*g* for 20 min followed by filtration of the supernatant through a 0.22 μM filter. His-tagged proteins were purified from spent tissue culture supernatant with either a 1 mL HisTrap HP column (GE Healthcare) using an ÄKTAxpress or ÄKTApure instrument (GE Healthcare) or a bespoke purification instrument for parallel protein purification [28] in a His MultiTrap HP 96-well plate (GE Healthcare).

### Western blotting

To determine protein integrity, 10 µL of transfection supernatant was resolved by SDS-PAGE using Novex NuPAGE 4–12% Bis Tris precast gels (Life Technologies) under reducing conditions, transferred to nitrocellulose membrane (Invitrogen), blocked with 2% BSA in phosphate-buffered saline (PBS)/0.1% Tween-20 (PBST), and probed with 0.02 µg/mL of streptavidin-HRP (Jackson Immunoresearch) diluted in PBS-2% BSA. After washing, biotinylated proteins were detected by addition of SuperSignal West Pico Chemiluminescent substrate (PIERCE) and developed on photographic film (Amersham Hyperfilm ECL, GE Healthcare).

### Enzyme-linked immunosorbent assay (ELISA)

ELISAs were performed by capturing biotinylated bait proteins into individual wells of streptavidin-coated 384-well plates (Greiner Bio-one). Plates were washed for 30 min with 50 μL PBS-T (0.2% Tween) and blocked with PBS-2% BSA for a minimum of 3 h. 20 μL of a bait protein diluted in PBS-2% BSA at a concentration previously determined as the amount required to saturate the biotin binding capacity of the well were added in triplicate and incubated for at least 16 h at 4 °C. Antisera were centrifuged at 13,000 rpm for a minimum of 1 h at 4 °C, diluted in PBS-2% BSA and incubated with rotation for at least 16 h at 4 °C before adding to the antigen-coated plates for 1 h. Serum dilutions used were: native Tanzanians 1:5000, native Malaysians 1:1000, imported malaria 1:500–1:1000, European travellers 1:100–1:500, and Malawian pooled sera resuspended to 20 mg mL^−1^ and used 1:1000. Plates were washed 3× in PBS-T before incubating with 1:10,000 dilution of peroxidase-conjugated AffiniPure goat anti-human IgA + IgG + IgM (H + L) (Jackson ImmunoResearch) in PBS-2%BSA for 1 h. Plates were washed in PBS-T and the HRP substrate ABTS (KPL) was added and absorption at 405 nm determined using an automated plate reader (FluoStar Optima, BMG Labtech).

### Data analysis

Data are available in Additional file 1 and all data analysis was performed in R (version 3.5.2). Background signals from a negative control well were subtracted from ELISA absorbance data, and because there was little signal to most antigens for each patient, the median across all antigens was used as a robust measure of background immunoreactivity. For cross-reactivity analysis, pairwise Spearman correlations between each measured antigen were calculated across patient sera. Immunoreactivity values that were negative after background subtraction were rounded to zero to avoid correlating uninformative negative signals. Significance tests for each rank correlation were corrected for multiple-testing using the Benjamini–Hochberg procedure. Based on the results of the cross-reactivity analysis, all antigens with significantly correlated immunoreactivity across different species were excluded from further analysis (*Pf*HPzz, *Pv*HPzz, *Pm*P41, *Pm*MSP5, *Pv*MSP5). Logistic regression models to predict exposure were computed using the generalized linear models “glm” function in R. One model was fitted per species, making a total of five classifiers. For every model, each patient was designated as either: diagnosed for that particular species (either by qPCR or microscopy); a negative control from a non-endemic region with no infection history; or, as ambiguous, if the patient lived in an endemic region, but was not diagnosed for that particular species. Ambiguous cases were omitted in model training, since they could not be classified reliably. Once labelled, the data sets were randomly divided approximately 50:50 into a testing set and training set. Logistic regression models were fit to the training set, then evaluated on the testing sets. Receiver operating characteristic (ROC) curves were calculated using the model predictions on the testing set and their known diagnosed labels. Curves were coloured by an arbitrary cost function, summing the number of false positives and false negatives at each threshold to highlight the relative optima [32, 33]. The area under the curve (AUC) was calculated using the PRROC package [34]. To determine a confidence-interval for the ROC curve estimates, the model fitting and evaluation process was repeated for 10 different random splits of the data into training and testing sets. Average true positive and false positive rates were calculated at each threshold, along with a 95% confidence interval from the 10 stratifications. When calculating model scores across all patients for every species the same procedure was followed except 100 instead of 10 random sub-samplings were used to ensure that every patient was included on at least one testing set after random splitting.

## Results

**Fig. 1:**
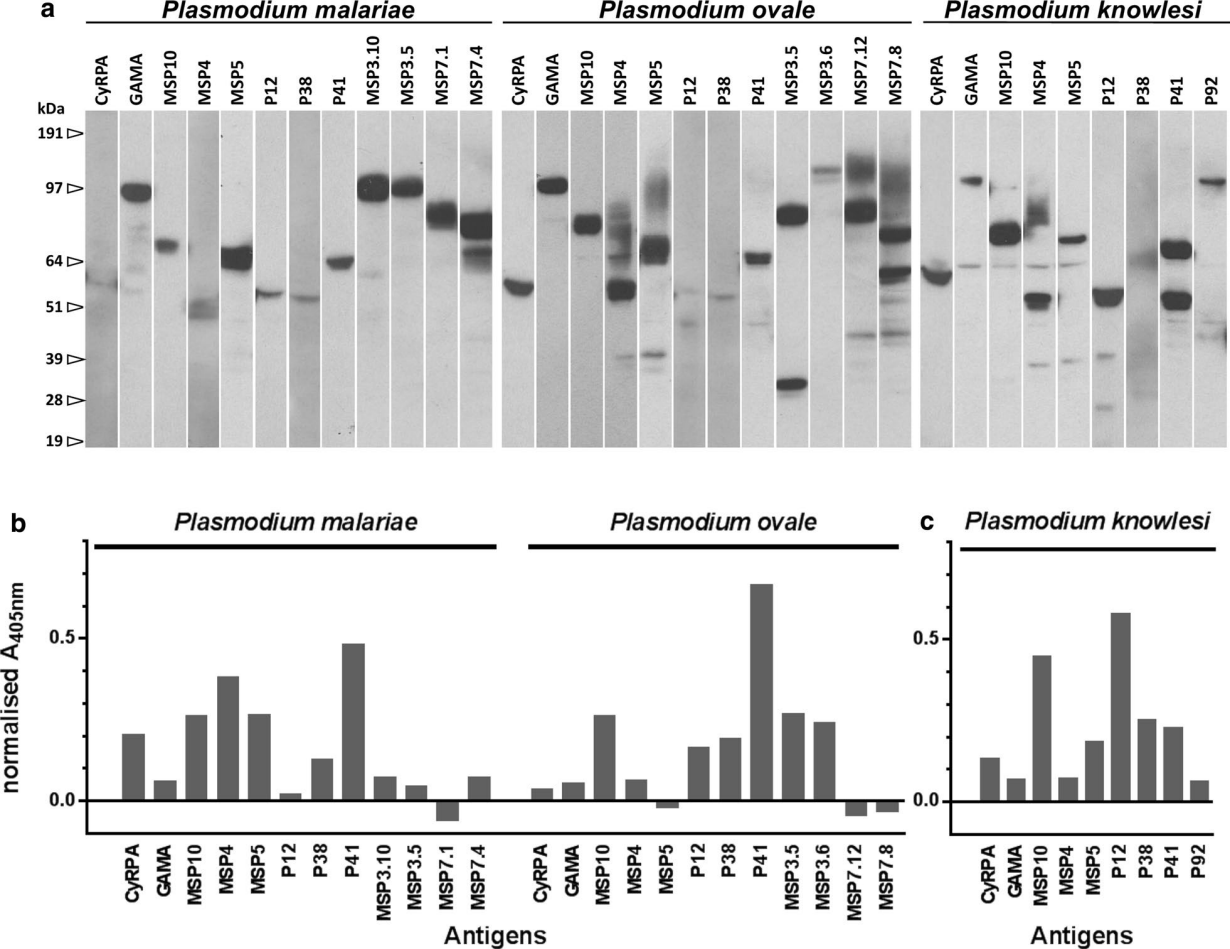
Immunoreactivity of recombinant *Plasmodium* antigens in sera from adults living in endemic regions. **a** Expression plasmids encoding the entire ectodomains of the proteins from the named species were expressed as enzymatically monobiotinylated soluble recombinant proteins in HEK293 cells. Spent tissue culture supernatant was normalized and resolved by SDS-PAGE under reducing conditions, blotted and detected by Western blotting using streptavidin-HRP. All proteins contain a C-terminal rat Cd4d3+ 4 and enzymatically biotinylatable tag. The named proteins from each species were enzymatically monobiotinylated during expression and captured on streptavidin-coated plates. Pooled sera from Malawian adults was used to probe the *P. malariae* and *P. ovale* proteins (**b**), and sera from 10 Malaysian individuals with PCR-confirmed single *P. knowlesi* infections were used for the *P. knowlesi* (**c**). Immunoreactivity was quantified by ELISA using an anti-human alkaline-phosphatase conjugated secondary antibody that hydrolyzed a substrate forming a product with maximal absorbance at 405 nm; responses were normalized to a control protein

With the aim of identifying antigens that could be used for serological markers of infection for *Plasmodium* parasites that infect humans, 12 proteins from *P. falciparum* and 10 proteins from *P. vivax* which were previously shown to be highly immunoreactive to sera from patients living in endemic regions were selected [12, 18] (Table 1). From these proteins, 8 that were produced at high levels in the expression system (CyRPA, GAMA, MSP10, MSP4, MSP5, P12, P38, P41) were selected to identify the orthologous proteins from the genome sequences of *P. knowlesi* [22]*, P. ovale* and *P. malariae* [23, 24] (Table 1). The P92 orthologue from *P. knowlesi* and 2 paralogues from both the MSP3 and MSP7 multigene family from *P. ovale* and *P. malariae* were also selected since orthologues of these proteins are highly immunoreactive in *P. falciparum* (Table 1). Protein expression plasmids encoding the entire ectodomains for the selected genes were made by gene synthesis, and proteins were expressed as soluble recombinant proteins by transfecting HEK293 cells. As expected, protein expression levels varied considerably, and most were expressed at usable levels at the expected size (Fig. 1a, Table 1). Exceptions included the P38 orthologue from all 3 species, P12 from *P. malariae* and *P. ovale,* and *Pm*CyRPA, which were all repeatedly expressed at low levels in independent transfections. *Po*MSP3.5, *Po*MSP7.8 and *Pk*P41 showed evidence of some proteolytic processing (Fig. 1a). In summary, orthologues of proteins in the genomes of *P. knowlesi*, *P. ovale* and *P. malariae* that are highly immunoreactive to sera from patients with *P. falciparum* and *P. vivax* infections were identified and expressed as soluble recombinant proteins for serological screening.

**Table 1:** Details of the *Plasmodium* spp. proteins expressed in this study

Sp.	Official nomenclature	Synonym/s	Accession number	Region expressed	Length (aa)	Exp. level	PlasmoDB, previous ID	Addgene ID
*P. falciparum*	MSP1*	MSA1, Pf190, Pf195	PF3D7_0930300	V20-S1701	1682	Low	Merozoite surface protein 1	47709
	AMA1*	Pf83, RMA1, PfAMA1	PF3D7_1133400	Q25-T541	517	High	Apical membrane antigen 1, PF11_0344	47741
	P92*	Pf92	PF3D7_1364100	A26-S770	745	Low	6-cysteine protein, PF13_0338	47728
	MSP4		PF3D7_0207000	Y29-S253	225	Low	Merozoite surface protein 4, PFB0310C	47717
	MSP5		PF3D7_0206900.1	N22-S251	230	Low	Merozoite surface protein 5, PFB0305C	47718
	P12*	Pf12, Pfs12	PF3D7_0612700	H26-S323	298	High	6-cysteine protein, MAL6P1, PFF0615C	47725
	GAMA	PSOP9	PF3D7_0828800	L22-P710	689	Med	GPI-anchored micronemal antigen	47747
	MSP10*		PF3D7_0620400	H27-S503	477	Med	Merozoite surface protein 10, MAL6P1.221, PFF0995C	47719
	P38	Pf38, Pfs38	PF3D7_0508000	Q22-S328	307	High	6-cysteine protein, PFE0395C	47727
	MSP3.1*	MSP3, SPAM	PF3D7_1035400	K26-H354	328	Med	Merozoite surface protein 3, PF10_0345	47731
	P41	Pf41, Pfs41	PF3D7_0404900	K21-S378	358	High	6-cysteine protein, PFD0240C	47739
	MSP7		PF3D7_1335100	T28-M351	324	Med	Merozoite surface protein 7, PF13_0197	47735
	HPzz*	PFA0210c	PF3D7_0104200	Y24-D466	443	Low	StAR-related lipid transfer protein, MAL1P1.33	50821
*P. vivax*	MSP4		PVX_003775	A26-S227	202	Low	Merozoite surface protein 4, putative, PV003775	68510
	MSP5*		PVX_003770	R22-S367	346	Med	Merozoite surface protein 5, PV003770	68511
	P12*		PVX_113775	F24-A339	316	Med	6-cysteine protein, PV113775	68516
	GAMA		PVX_088910	L21-S749	729	Med	GPI-anchored micronemal antigen, putative, PV088910	68522
	CyRPA		PVX_090240	T23-D366	344	Med	Cysteine-rich protective antigen, putative, PV090240	68525
	HPzz*		PVX_081550	R23-F495	473	Low	StAR-related lipid transfer protein, putative, PV081550	68532
	P38*		PVX_097960	K29-G334	306	Med	6-cysteine protein, PV097960	68518
	MSP7.1*		PVX_082700	E22-Y420	399	Med	Merozoite surface protein 7 (MSP7), PV082700	68512
	MSP3γ	MSP3.1	PVX_097670	N21-K845	825	Med	Merozoite surface protein 3, PV097670	68506
	P41*		PVX_000995	E22-E384	363	Med	6-cysteine protein, PV000995	68519
*P. malariae*	CyRPA		PmUG01_05040800	E20-D358	339	Low	Cysteine-rich protective antigen, putative	126817
	GAMA		PmUG01_05017200	L22-S764	743	High	GPI-anchored micronemal antigen	126818
	MSP10*		PmUG01_11043200	N27-S389	363	Low	Merozoite surface protein 10, putative	126819
	MSP4		PmUG01_04025600	Y29-S206	178	Low	Merozoite surface protein 4, putative	126820
	MSP5*		PmUG01_04025700	K22-L291	270	High	Merozoite surface protein 5	126821
	P12	P12p	PmUG01_11050400	Y19-S333	315	Low	6-cysteine protein	126822
	P38		PmUG01_06016900	M1-A310	310	Low	6-cysteine protein, putative	126823
	P41*		PmUG01_03015000	Q23-D374	352	Med	6-cysteine protein, putative	126824
	MSP3.10		PmUG01_06022000	K24-I525	522	High	Merozoite surface protein 3, putative	126825
	MSP3.5		PmUG01_06022600	N21-S562	542	High	Merozoite surface protein 3, putative	126826
	MSP7.1		PmUG01_12029500	R25-I442	418	High	MSP7-like protein, putative	126827
	MSP7.4		PmUG01_12029900	K22-V394	373	High	MSP7-like protein, putative	126828
*P. ovale*	CyRPA		POVCU1_054880	S20-D359	340	Med	Cysteine-rich protective antigen, putative, SBS99762.1	126829
	GAMA		PocGH01_05012100	L22-S755	734	High	GPI-anchored micronemal antigen, putative	126830
	MSP10*		PocGH01_11036900	Y27-S430	404	High	Merozoite surface protein 10, putative	126831
	MSP4		PocGH01_04023000	N26-S209	184	High	Merozoite surface protein 4, putative	126832
	MSP5		PocGH01_04023100	F20-S311	292	High	Merozoite surface protein 5, putative	126833
	P12	P12p	PocGH01_11044100	E23-S301	279	Low	6-cysteine protein	126834
	P38		PocGH01_10033500	K25-S332	308	Low	6-cysteine protein, putative	126835
	P41*		PocGH01_03012400	E16-E375	360	Med	6-cysteine protein, putative	126836
	MSP3.5*		PocGH01_10038700	K24-P480	457	Med	Merozoite surface protein 3, putative	126837
	MSP3.6		PocGH01_10038800	K24-S732	709	Low	Merozoite surface protein 3, putative	126838
	MSP7.12		PocGH01_12028800	K23-T441	419	Med	MSP7-like protein, putative	126839
	MSP7.8		PocGH01_12028400	K23-T379	357	Med	MSP7-like protein, putative	126840
*P. knowlesi*	CyRPA		PKNH_0515800	N23-E366	344	Med	Cysteine-rich protective antigen, putative, PKH_052740	126841
	GAMA	PSOP9	PKNH_1322900	L21-S700	680	Med	GPI-anchored micronemal antigen, putative, PKH_050210	126842
	MSP10*		PKNH_1129800	N27-S417	391	High	Merozoite surface protein 10, putative, PKH_112880	126940
	MSP4		PKNH_0414100	D26-S182	157	Med	Merozoite surface protein 4, putative, PKH_041300	126843
	MSP5		PKNH_0414200	H22-S354	333	Med	Merozoite surface protein 5, PKH_041310	126844
	P12*		PKNH_1137300	F24-S323	300	Med	6-cysteine protein, PKH_113620	126845
	P38*		PKNH_1025600	K3-S367	365	Low	6-cysteine protein, PKH_102490	126846
	P41		PKNH_0303000	E22-E393	372	High	6-cysteine protein, PKH_030970	126847
	P92		PKNH_1107200	D24-P857	834	Med	6-cysteine protein, PKH_110660	126848

Protein expression plasmids encoding the named genes from the five *Plasmodium* species that infect humans are detailed. ‘Region expressed’ refers to the ectodomain regions between the predicted signal sequence peptide and the transmembrane or GPI anchor sequence (if present). Expression levels are given as a guide only given the significant batch-to-batch variability observed using this approach and grouped into ‘high’ (between 5 and 50 μg/mL), ‘medium’ (0.5–5 μg/mL) and ‘low’ (0.005–0.5 μg/mL). Those proteins selected for further serological analysis are marked with an asterisk

A smaller subset within the expanded panel of recombinant proteins which were immunoreactive needed to be identified as suitable serological markers of infection. To determine which of the *P. ovale* and *P. malariae* proteins were immunoreactive, the responses to IgG, IgM and IgA immunoglobulins were tested using sera pooled from over 800 adults living in Malawi [19] where there is active transmission for both species [35]. For *P. malariae*, P41, MSP5, and MSP10 were selected as they were the most immunoreactive and expressed at acceptable levels (Fig. 1b); MSP4 was not selected because although immunoreactive, was repeatedly expressed poorly. Using the same criteria, the *P. ovale* proteins P41, MSP3.5 and MSP10 were selected (Fig. 1b). To identify the most suitable antigens for *P. knowlesi*, the immunoreactivity to the panel of proteins was tested using sera derived from 10 Malaysian patients with PCR-confirmed single *P. knowlesi* infections, and P12, P38 and MSP10 were chosen (Fig. 1c). From these experiments, a panel of 22 proteins were selected that contained 7 proteins from *P. falciparum*, 6 from *P. vivax*, and 3 from each of *P. malariae*, *P. ovale* and *P. knowlesi* (Table 1).

**Fig. 2:**
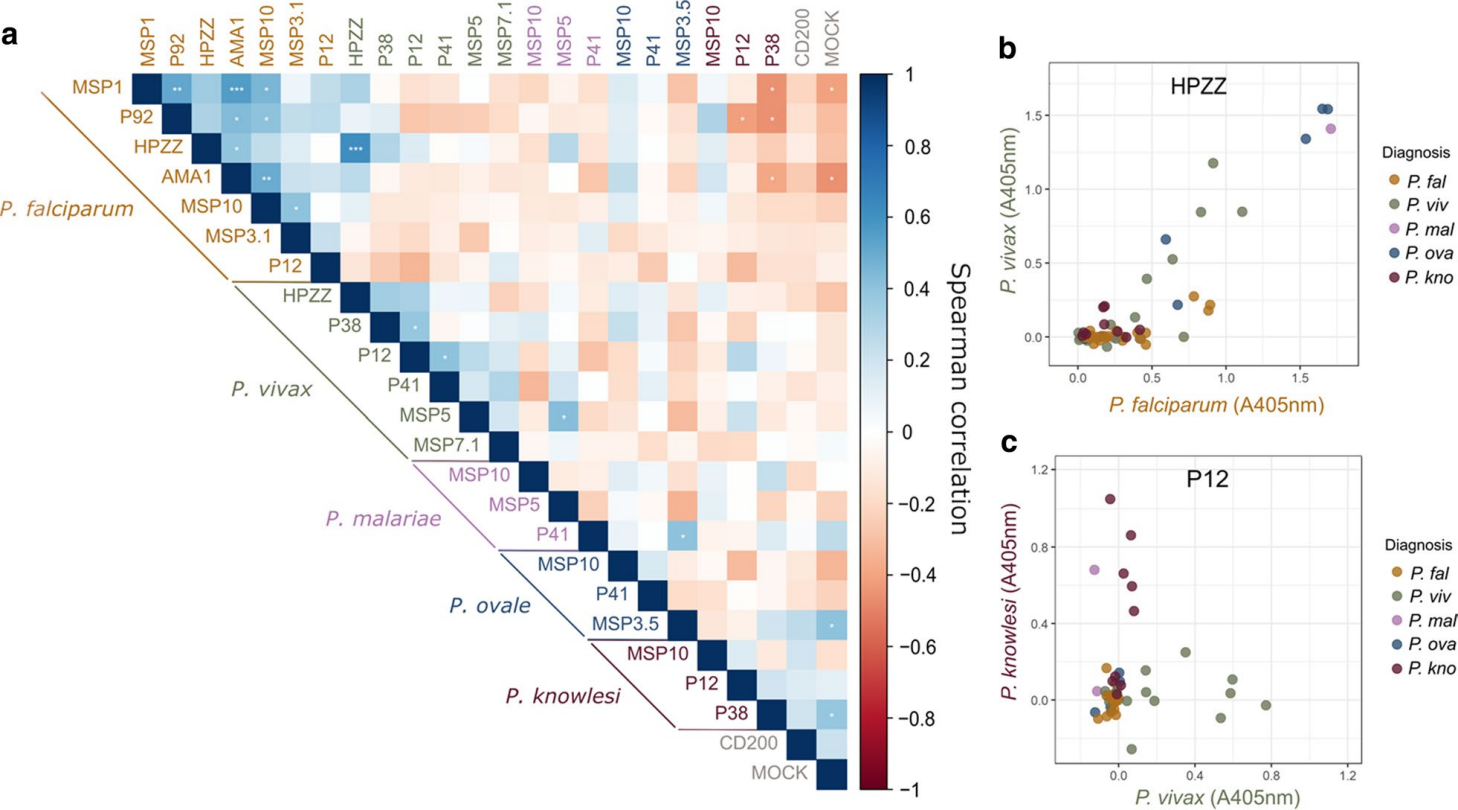
Intra-species correlations and cross-species reactivities to serological responses to recombinant *Plasmodium* spp. antigens. The immunoreactivities of the named antigens from each *Plasmodium* species to diagnosed patient sera were quantified by ELISA and their pairwise correlation determined (**a**). For *P. falciparum*, *P. vivax*, *P. ovale* and *P. malariae* infections, sera were obtained from Swedish travellers to malaria-endemic regions increasing the chances of a mono-infection; *P. knowlesi* infections were from Malaysian patients. Spearman correlations for each antigen pair show some expected clusters of correlations within a species, especially for *P. falciparum* and *P. vivax* where more samples were available, and potential cross-reactivity between antigens across species were identified. **b** Serological responses to *P. falciparum* and *P. vivax* HPzz orthologues are likely to be cross-reactive despite low sequence identity between orthologues. The immunoreactivity to the *P. falciparum* and *P. vivax* HPzz antigens are plotted for each patient with their diagnosis indicated. **c** Responses to *P. knowlesi* and *P. vivax* P12 orthologues show no evidence of cross-reactivity despite high primary protein sequence identity between orthologues. p values from a t-distribution significance test are indicated by asterisks. *p < 0.05; **p < 0.01; ***p < 0.001

Using this panel of 22 immunoreactive proteins, the antibody responses to a specific antigen from a particular *Plasmodium* species were examined to determine whether there was any detectable cross-reactivity with antigens from other species. Ideally, this would make use of sera from human patients which have diagnosed mono-infections for each *Plasmodium* species. To increase the chances that patients had been exposed to a single species, serum samples were obtained from patients of European origin who had contracted malaria by visiting malaria endemic regions and in which the infecting *Plasmodium* species had been confirmed by PCR. For the more common parasites, a reasonable number of samples were obtained: *P. falciparum* (*n* = 26), *P. vivax* (*n* = 17), but access to serum samples of travellers’ malaria with diagnosed infections for the rarer parasites was limited: *P. ovale* (*n* = 7), *P. malariae* (*n* = 3). For *P. knowlesi*, responses in the 10 Malaysian patients with PCR-confirmed single *P. knowlesi* infections were used. These patient sera were screened against the panel of all 22 antigens from five *Plasmodium* species. Two patients exhibited a broad reactivity to antigens from two species: a Swedish traveller diagnosed with *P. vivax* but whose serum exhibited broad reactivity across antigens from three *Plasmodium* species (*P. vivax*, *P. falciparum*, *P. knowlesi*) and one of the Malaysian patients diagnosed with *P. knowlesi* but whose serum reacted strongly with four out of six *P. vivax* antigens; this patient was subsequently identified as a migrant worker from Indonesia where *P. vivax* is endemic. Because it is likely that these patients had prior exposure to another *Plasmodium* species other than their diagnosed infection, these patients were removed from this analysis. The pairwise correlation of the responses to each of the antigens in the panel was examined in the remaining patients (Fig. 2a). For *P. falciparum* and *P. vivax*, where more samples were available, the antibody responses to most antigens within that species were positively correlated, as expected (Fig. 2a). There were two antigens that showed significant cross-reactivity between their orthologues: the HPzz proteins from *P. falciparum* and *P. vivax*, and MSP5 from *P. vivax* and *P. malariae.* Furthermore, responses to *P. malariae* P41 correlated with *P. ovale* MSP3.5. This suggested that responses to these proteins may not be suitable to diagnose exposure for these species, and so were omitted from further analyses. There was no evidence that sequence identity between orthologous proteins correlated with cross reactivity. For example, the amino acid sequence identity between *P. falciparum* and *P. vivax* HPzz proteins was only 39%, and yet responses showed strong evidence of cross-reactivity (Fig. 2b). By contrast, the sequence identity between *P. knowlesi* and *P. vivax* P12 was much higher (72%), and yet patient responses showed little evidence of cross-reaction (Fig. 2c). While this analysis must come with the caveat that the number of serum samples from travellers with malaria is limited, especially for the rarer parasites, *P. ovale* and *P. malariae*, antigens that appeared potentially cross-reactive between species were excluded, and this apparent cross-reactivity did not correlate with sequence identity between the orthologous proteins.

To determine whether the panel of proteins could be used in serological assays to establish exposure to different human-infective *Plasmodium* species, a larger panel of serum samples from individuals with diagnosed infections that corresponded to both ongoing acute cases of imported malaria (*n *= 81), and endemic malaria from a cohort of Tanzanian adults (*n *= 21) were tested. For *P. knowlesi*, sera from the 10 patients diagnosed with *P. knowlesi* infections from Kapit Hospital in Malaysian Borneo were used. The antibody responses between the different sources were investigated by segregating the samples into three different categories: travellers of European origin (*n* = 53), travellers with an origin in a malaria-endemic area (*n* = 28), and individuals residing in an endemic area (*n* = 31). The responses to each antigen corresponding to the species diagnosis was plotted within each category, and while differences between the different patient groupings were observed on a per antigen basis (Additional file 2: Fig. S1a), when averaged across the panel, no systematic differences were observed (Additional file 2: Fig. S1b). The converse analysis was performed whereby the responses to the antigens corresponding to the species other than that in the diagnosis were plotted, and again segregated according to the different categories. As expected, there was little response across the antigen panel, although it was clear that the Tanzanian endemic samples showed evidence of exposure to other species other than the one for which they had a diagnosed ongoing infection, especially for *P. falciparum*, but also *P. ovale* (Additional file 2: Fig. S1c). Immunoreactivity to both HPzz orthologues from both *P. falciparum* and *P. vivax* and *Pf*MSP1 were observed which suggested antigen cross-reactivity, as had been already established for HPzz (Fig. 2), or, in the case of *Pf*MSP1, there was a higher background signal, including from the uninfected controls (Additional file 2: Fig. S1c).

A logistic-regression classifier was trained to combine the immunoreactivities to the antigens from each species into a prediction of prior exposure. Sera from both travellers and individuals living in endemic regions for each species were used together with unexposed control samples and iteratively and randomly split 50:50 into training and testing sets, with approximately equal representation of diagnoses for each species. For each *Plasmodium* species, a separate model was trained to assign a binary outcome indicating if a patient was infected by that species or not. A score near zero indicates that the model assigns a low probability of the patient being infected with the given *Plasmodium* species, while scores near one indicate likely infections. Because patients from endemic regions are very likely to have had prior exposure to species other than the one they were actively diagnosed with, the positive training set was defined as patients only with a confirmed species diagnosis by microscopy or PCR.

**Fig. 3:**
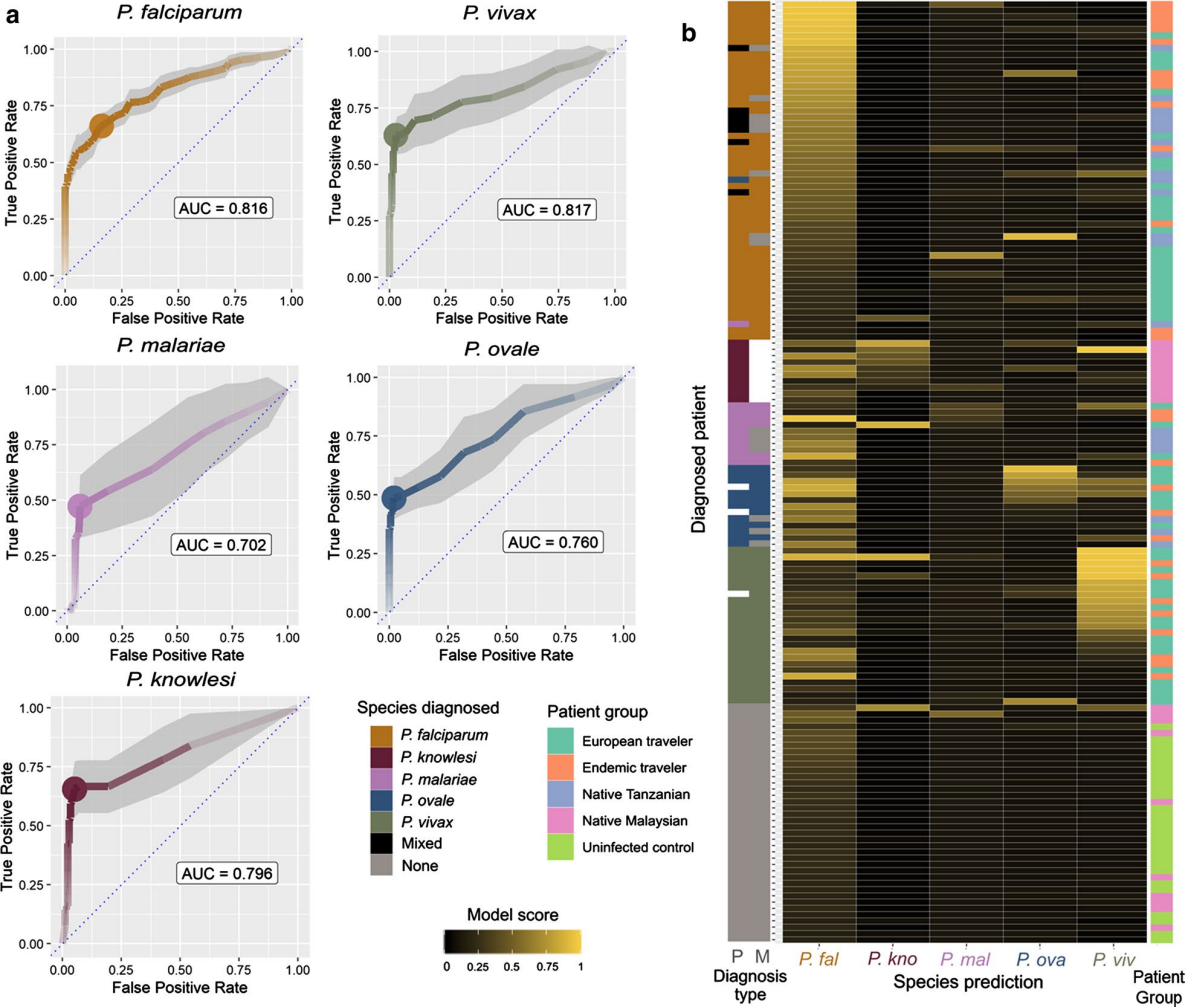
Classification of exposure to five human-infective *Plasmodium* species using a panel of recombinant *Plasmodium* proteins. **a** Receiver operating characteristic (ROC) curves of the performance of logistic regression models using all non-cross-reactive antigens for each species. Immunoreactivity data were randomly halved into training and testing sets, to fit the model and calculate ROC curves respectively; this process was repeated ten times to estimate a 95% confidence interval (grey shading). The threshold giving the maximum Youden’s index performance is marked with a dot. **b** Diagnostic scores from each species’ classifier for each patient and control sample. The classifications of 100 models from randomly split training and testing data were averaged. Brighter yellow indicates higher confidence in a positive diagnosis, according to the scale indicated. Diagnosis type is indicated as P: PCR; M: microscopy

The performance of each diagnostic model was evaluated, and the random sampling procedure into training and testing sets was iteratively repeated. To quantify the models’ performance, the averaged receiver operating characteristic (ROC) curves for predicting exposure to each species (Fig. 3a) was first calculated. Moderate predictive power (area under the curve (AUC) ≥ 0.7) was achieved for all species, and especially for *P. vivax, P. falciparum* and *P. knowlesi* where exposure could be predicted with good accuracy (AUC ≥ 0.8). The relatively small number of available diagnosed samples for the rarer parasites *P. ovale* (*n *= 18) and *P. malariae* (*n *= 17), limited the performance of their classifiers, as well as in the case of *P. malariae*, the reliance on just a single antigen (*Pm*MSP10) after removing those that showed evidence of cross-reactivity.

Diagnostic models were used to determine a likelihood of prior exposure of the patients to each of the different *Plasmodium* species (Fig. 3b). In general, the models returned a positive prediction for the parasite species that agreed with the diagnosis, and the sera from unexposed controls being negative. The classifiers performed less well on those patients with co-infections and those diagnosed by PCR rather than microscopy (Fig. 3b). As expected, several individuals showed strong evidence of prior exposure to a different *Plasmodium* parasite species other than the one for which they were positively diagnosed. For example, some individuals positively diagnosed with *P. ovale* infections, also showed evidence of prior exposure to *P. falciparum* and *P. vivax*.

**Fig. 4:**
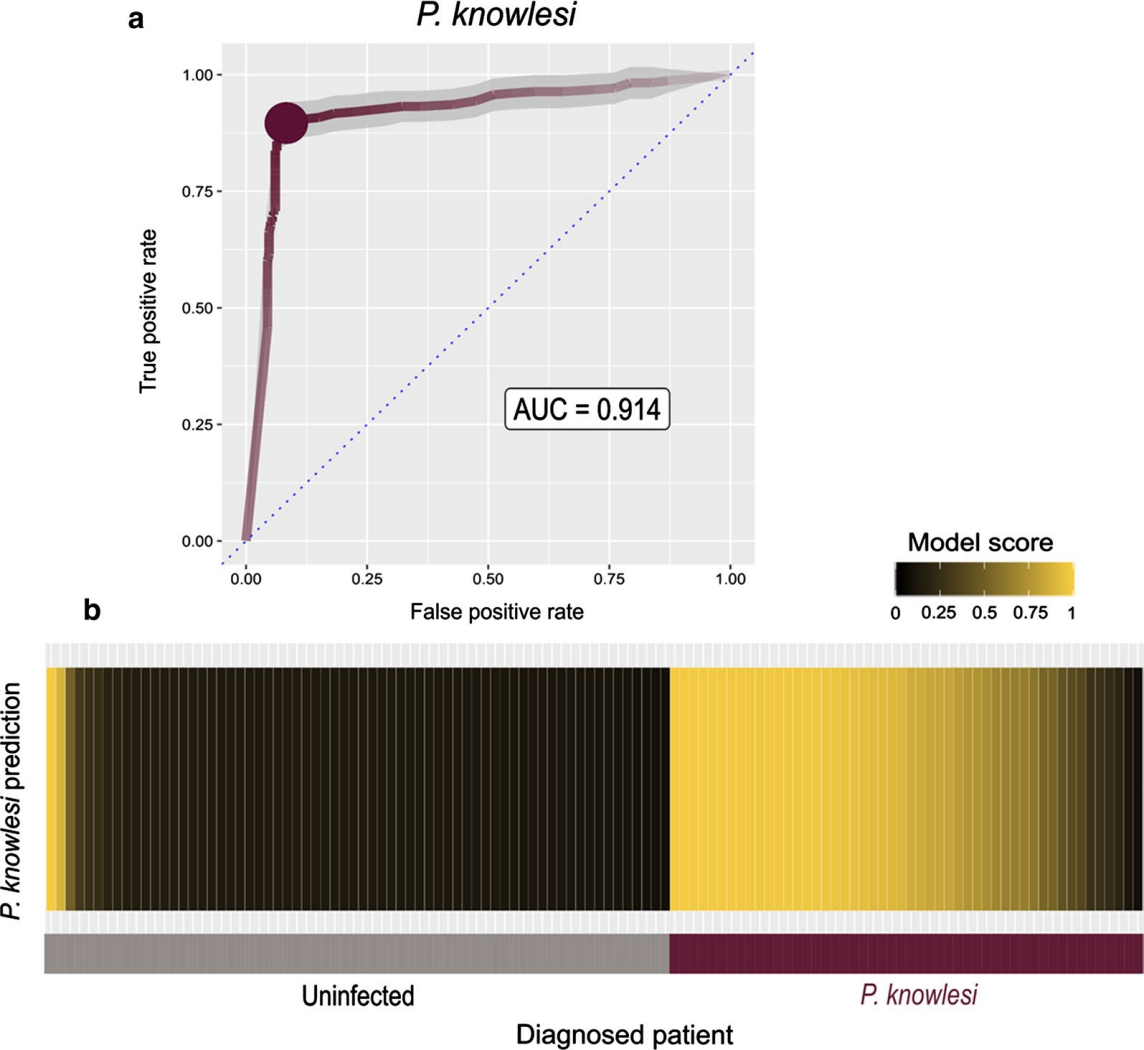
Accurate serological diagnosis of *Plasmodium knowlesi* infections using a small panel of recombinant antigens. **a** ROC curve for a logistic regression model combining signal from three *P. knowlesi* antigens. The shaded 95% confidence interval is estimated from ten random sub-samplings of the data into model training and testing sets. The threshold giving the maximum Youden’s index performance is marked with a dot. **b** Average model confidence in *P. knowlesi* exposure for each patient (colour-coded along the x-axis: grey: uninfected controls, burgundy: *P. knowlesi*-infected diagnosed patient) after 100 rounds of sub-sampling into testing sets

Following the encouraging performance of the antigen panel in diagnosing infections by *P. knowlesi*, a larger number of serum samples comprising 50 *P. knowlesi* malaria patients and 66 uninfected controls from the same region in Malaysia were evaluated. Using the logistic regression model to combine the responses against all three antigens (*Pk*MSP10, *Pk*P12, *Pk*P38), *P. knowlesi* infections could be identified with high confidence (AUC > 91%) (Fig. 4a). If a threshold is set to a model score of above 0.5, then 82% of *P. knowlesi*-infected patients are correctly diagnosed at a false-positive rate of 3% (Fig. 4b).

## Discussion

Malaria continues to be a significant global health problem although cases have dropped in recent years due to the deployment of effective intervention measures [1]. Spurred on by these successes, the malaria control policies of governments and international agencies are moving towards the goal of eradication, which will require more sensitive detection and tracking of the different species in endemic regions. The application of serological screening may have an increasingly important role as transmission declines because the longevity of antibody responses should provide a historical record of exposure rather than being limited to the detection of current infections [36]. In areas of declining transmission, where there are increasing incidences of sub-microscopic infection, models using data from serological surveillance have been shown to provide precise estimates of parasite transmission [10]. Because of the asexual amplification of malaria parasites in the blood, those antigens expressed during these blood stages are likely to be good choices for diagnostic antigens because they are known to be particularly immunodominant. Several serological assays have been developed to detect exposure to *Plasmodium* parasites although few have attempted to distinguish infections by the different species [37]. This has mainly been due to the lack of a genome sequence from the two parasites, *P. ovale* and *P. malariae,* making the cloning and expression of many candidate proteins from these species difficult. Recent sequencing of *P. ovale* and *P. malariae* [23, 24] has now permitted the expression of several candidate proteins from these parasites. Here, a panel of antigens was compiled that are likely to be expressed by the blood stages using the recent availability of the genome sequences of all five main species of *Plasmodium* parasites that infect humans, together with the approach of expressing extracellular parasite proteins in a functionally active form using a mammalian expression system. After removing those proteins which showed evidence of cross-reactive responses, it was found that immunoreactivity to the antigens from *P. ovale*, (MSP10, P41), *P. malariae* (MSP10) and *P. knowlesi* (MSP10, P12, P38), together with logistic regression modelling had moderate power to predict prior exposure to these species, and performed especially well for *P. knowlesi*.

Expressing *Plasmodium* proteins is known to be technically challenging, possibly due to the unusual codon bias or highly repetitive amino acid sequences often found in *Plasmodium* proteins [15, 38]. Most studies expressing recombinant antigens for serological assays use prokaryotic expression systems (especially *Escherichia coli*) or cell-free systems, such as wheat germ extracts [39–41]. While these expression systems have the advantages of being cost effective, high yielding and widely available, they may not be suitable for expressing extracellular proteins because they usually require a reducing environment which would interfere with the formation of structurally critical disulfide bonds [16, 42]. While protein refolding procedures can be used, they are usually complex, time consuming, and have uncertain outcomes which are often hard to determine if they have been successful [43]. When used for serological screening, proteins that do not adopt the native conformation are unlikely to be useful in detecting antibodies that recognise conformational epitopes, potentially reducing sensitivity. By using a mammalian expression system to produce *Plasmodium* proteins, it was previously shown that these recombinant proteins can retain conformational epitopes, and shown that for the majority of antigens, a large fraction of the immunoreactivity to sera from *Plasmodium*-exposed patients is heat labile, demonstrating the benefit of using proteins that retain native folding for serological assays [16]. *Plasmodium* blood-stage proteins expressed using this approach have been useful in sero-epidemiological studies for *P. falciparum* [12, 44] and *P. vivax* [8]. One likely consequence of using conformational epitopes in serological assays, which are aimed at distinguishing between species, is that the percentage sequence identity between orthologous proteins is unlikely to be a reliable indicator of cross-reactivity. In the current study, a likely cross-reactivity was observed between the *P. falciparum* and *P. vivax* orthologues of the HPzz protein between patients diagnosed with these parasites, despite there being only ~ 40% amino acid shared sequence identity. By contrast, very little cross-reactivity was observed in patient serological responses between the *P. vivax* and *P. knowlesi* P12 orthologues which are relatively well conserved, sharing > 70% amino acid sequence identity. One likely possibility is that the informative epitopes for these proteins are mainly composed of the precise arrangement of solvent-exposed amino acids, making overall linear sequence identity less important, whereas repeats of just a few amino acids, for example the repetitive “NANP” motifs in the circumsporozoite protein can be highly immunogenic [45].

A panel of three recombinant antigens (*Pk*MSP10, *Pk*P12, *Pk*P38), was found to accurately detect exposure to *P. knowlesi* using a serological assay. This compares well with a recent study where the authors also selected candidates that were orthologous to known immunoreactive proteins in *P. falciparum*, and selected four antigens that were associated with *P. knowlesi* exposure [46]. Together, these studies will contribute towards the further development of accurate serological assays for this parasite which is becoming an increasing public health concern in Southeast Asia, particularly in Malaysia where it is now replacing *P. falciparum* and *P. vivax* as the major cause of malaria [47, 48].

The use of plasma from native Europeans who had contracted travellers’ malaria and had been diagnosed accurately increased the chances that they had only been exposed to a single species which was useful for establishing which responses to different antigens might be cross reactive. However, accessing large numbers of these samples was difficult, especially for the rarer parasites, *P. malariae* and *P. ovale,* and therefore it is not certain that these patients had been previously exposed to other species of *Plasmodium*. Together with other assays that have been described for serological diagnosis of *Plasmodium* infections [37, 46, 49], this panel of proteins will contribute to an assay that will be a useful tool for establishing prior exposure to different species of *Plasmodium* parasites. Surveying more patient samples in longitudinal cohorts in a variety of transmission settings will be necessary to establish whether the responses to the proteins described here are different in patients living in different endemic regions, and vary according to the age and exposure of the patient.

## Conclusions

The recent availability of genome sequences for *Plasmodium* parasites was used to create a panel of recombinant proteins corresponding to immunoreactive blood stage proteins from five human-infective *Plasmodium* species. This panel of proteins will provide a basis for developing serological assays to determine exposure to the different species of parasite for serological surveillance and diagnostics.

## Supplementary information

**Additional file 1:** Datasets used for the analysis. The ELISA absorbance values used for the data analysis are provided as separate spreadsheets. Patient groups where applicable are numbered: 1—European traveller; 2 and 3—Imported malaria; 4—Native Tanzanian; 5—Control group; 6—Native Malaysian.

**Additional file 2: Fig. S1.** Antibody responses to the *Plasmodium* antigen panel across sample groups. (**a**) Immunoreactivity to antigens for patients diagnosed with an infection from the corresponding species, grouped by patient background. (**b**) Average immunoreactivity to antigens for each species in patients diagnosed for that species. (**c**) Background immunoreactivity to antigens for patients *not* diagnosed with the corresponding *Plasmodium* species.

## Data Availability

All plasmid protein expression constructs are available from the Addgene resource provider (www.addgene.org). The datasets used and/or analysed during the current study are available in Additional file 1.

## Abbreviations

AUC: area under curve
ELISA: enzyme-linked immunosorbent assay
HEK: human embryonic kidney
PCR: polymerase chain reaction
RDT: rapid diagnostic test
ROC: receiver operator characteristic
SDS PAGE: sodium dodecyl sulfate polyacrylamide gel electrophoresis
