# Supplementary figures and images for: A panel of recombinant proteins from human-infective Plasmodium species for serological surveillance
Source: Malar J. 2020 Jan 17;19:31. doi: 10.1186/s12936-020-3111-5 (PMC6969409; doi:10.1186/s12936-020-3111-5)

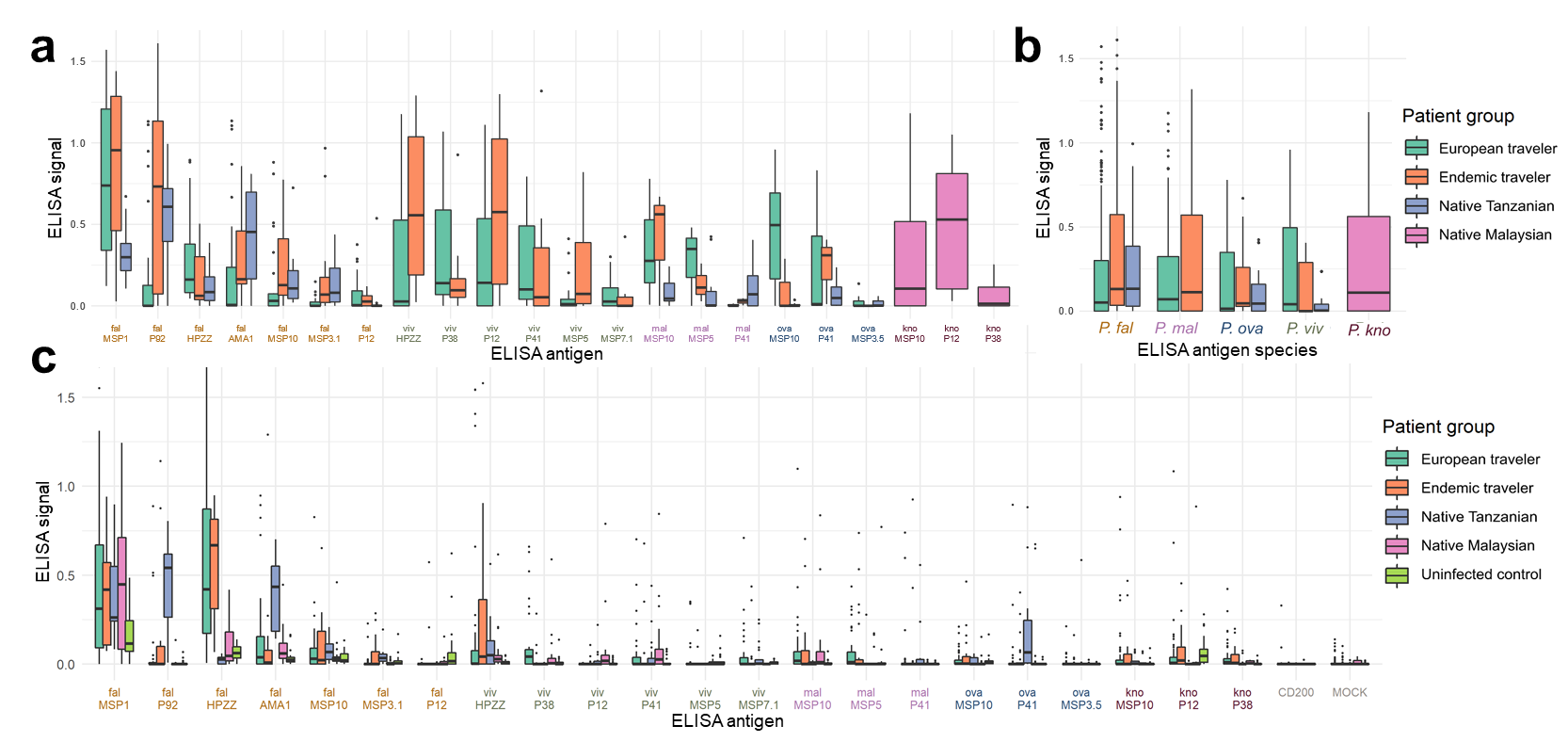

Supplement: Supplementary file 2 — Additional file 2: Fig. S1. Antibody responses to the Plasmodium antigen panel across sample groups. (a) Immunoreactivity to antigens for patients diagnosed with an infection from the corresponding species, grouped by patient background. (b) Average immunoreactivity to antigens for each species in patients diagnosed for that species. (c) Background immunoreactivity to antigens for patients not diagnosed with the corresponding Plasmodium species. [file 12936_2020_3111_MOESM2_ESM.png]
